# Validation of the TruBlue Light Laser for Laryngeal Somatosensory and Perturbation Testing

**DOI:** 10.1002/lary.70585

**Published:** 2026-04-25

**Authors:** Adrianna C. Shembel, Ted Mau, Youri Maryn, Elizabeth Young, Julianna Smeltzer, Lesley Childs, Shumon Dhar

**Affiliations:** ^1^ School of Behavioral and Brain Sciences, University of Texas at Dallas Richardson Texas USA; ^2^ Callier Center for Communication Disorders Dallas Texas USA; ^3^ Department of Otolaryngology‐Head and Neck Surgery University of Texas Southwestern Medical Center Dallas Texas USA; ^4^ Division of Laryngology, Department of Otolaryngology – Head and Neck Surgery Stanford University School of Medicine Palo Alto California USA; ^5^ Phonanium Antwerp Belgium; ^6^ European Institute for ORL‐HNS Wilrijk Belgium; ^7^ Department of Rehabilitation Sciences, Faculty of Medicine and Health Sciences Ghent University Ghent Belgium

**Keywords:** blue light laser, fundamental frequency variability, laryngeal perturbation, laryngeal protective closure reflex, laryngeal somatosensation

## Abstract

**Objectives:**

Clinical assessment of laryngeal somatosensation is limited by the lack of precise tools to directly stimulate the larynx and quantify sensorimotor responses. This study validated a blue light laser method for laryngeal somatosensory testing in vocally healthy adults and developed acoustic measures to quantify vocal responses to controlled laryngeal perturbations.

**Methods:**

In this prospective validation study, a 445‐nm diode blue light laser was delivered through a channeled flexible laryngoscope to the arytenoid mucosa. Single subablative pulses (1–10 W, 30 ms) were applied during quiet breathing or sustained phonation to determine perceptual and reflexive sensory thresholds, discrimination acuity, and laryngeal perturbation responses. Acoustic recordings during perturbations were analyzed in Praat using custom software to extract continuous fundamental frequency variability and quantify perturbation magnitude and recovery.

**Results:**

Participants demonstrated reliable perceptual detection and laryngeal reflexive responses to laser stimulation. Mean perceptual sensory threshold was 1.46 W (SD = 1.17) and mean laryngeal response threshold was 4.62 W (SD = 2.04). Sensory ratings were higher during stimulation than foil trials (*p* < 0.001). Sensory perception increased with stimulation intensity (~0.3 points per 1 W; *p* < 0.001), and higher wattage increased odds of eliciting a laryngeal response (OR = 1.38; *p* < 0.001). Discrimination accuracy averaged 78.5% (OR = 1.45; *p* = 0.002). Laser stimulation during phonation produced measurable acoustic perturbations (peak SD(*f*_0_) = 19.35 Hz; recovery = 0.48 s).

**Conclusion:**

Blue light laser stimulation is a feasible and precise method for evaluating laryngeal somatosensation and vocal sensorimotor responses.

**Level of Evidence:**

3.

## Graphical abstract

This study validates a blue light laser method for assessing laryngeal somatosensory function by measuring perceptual thresholds, reflexive responses, discrimination acuity, and vocal perturbations in healthy adults. Results demonstrate that the method provides precise, reliable stimulation and yields quantifiable sensory and acoustic responses, including graded perception, reflex activation, and measurable perturbations in fundamental frequency. Overall, blue light laser stimulation is a feasible, low‐risk, and clinically promising tool for evaluating laryngeal sensorimotor function.

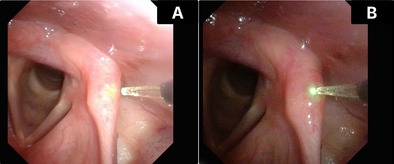

## 1 Introduction

Somatosensory feedback from the larynx plays a central role in airway protection, phonatory stability, regulation of vocal effort and fatigue, and rapid compensation to perturbation [1, 2, 3, 4, 5, 6, 7, 8]. Despite its importance, methods for quantifying laryngeal somatosensory function remain limited. Current approaches, including air puffs [9, 10, 11], and aesthesiometers [12, 13, 14] delivered through a channeled laryngoscope, provide relatively coarse and variably delivered stimuli [15, 16, 17]. These limitations constrain testing of sensory thresholds, discrimination acuity, and reflexive motor responses with sufficient precision to study laryngeal sensorimotor control.

Sensory threshold testing provides information about both conscious sensory detection and reflexive laryngeal motor responses. Discrimination testing assesses the ability to detect differences in stimulus intensity and reflects the resolution of afferent laryngeal feedback. Perturbation paradigms examine how the laryngeal motor system responds and adapts to brief, unexpected somatosensory input during phonation. Alterations in these domains have been implicated in disorders such as primary muscle tension dysphonia [15], Parkinson's disease [18, 19], irritable larynx syndrome [20], and chronic refractory cough [21, 22]. However, existing tools lack the precision and reproducibility needed to clearly define sensorimotor abnormalities in these disorders.

Blue diode laser stimulation provides a more controlled approach to direct laryngeal stimulation. Targeted stimulation of the arytenoid region evokes laryngeal airway protection reflexes (e.g., brief and ballistic vocal fold adduction) [9, 23], which can be used to probe laryngeal somatosensory responses and sensorimotor function. Stimulation with the blue light laser likely produces a localized photothermal mucosal stimulus capable of activating superior laryngeal afferents and evoking protected laryngeal motor responses. Because the stimulus is delivered as localized thermal energy, it may preferentially activate superficial thermo‐ or nociceptive afferents, although contributions from polymodal or mechanosensitive afferents cannot be excluded. Output can be adjusted through wattage and pulse duration, allowing delivery of finely graded, subablative thermal stimuli spanning subthreshold to suprathreshold levels. Unlike contact‐based methods (e.g., aesthesiometers), laser stimulation reduces variability related to force application, mucosal deformation, and angle of instrumentation.

Furthermore, traditional acoustic measures such as jitter, shimmer, and cepstral metrics are poorly suited to capture the rapid, transient vocal changes that follow direct laryngeal stimulation, as they rely on averaged phonatory segments. More temporally sensitive analyses are required to characterize response latency, magnitude, and recovery dynamics.

Improved precision in both stimulus delivery and response measurement is necessary to advance mechanistic models of laryngeal sensorimotor control and to better define the role of somatosensory dysregulation in voice and upper airway disorders. To address current limitations in laryngeal sensory testing, the primary objective of this study was to validate a blue light laser procedure for assessing perceptual and reflexive laryngeal sensory thresholds, discrimination acuity, and vocal responses to laryngeal perturbations in vocally healthy individuals. The secondary objective was to develop acoustic methods to quantify these laryngeal perturbations.

## 2 Material and Methods

The study design was a prospective observational validation study with experimental stimulus delivery in healthy participants.

### 2.1 Participants

The study was approved by the UT Southwestern Institutional Review Board (IRB: STU‐2020‐0720). Fifty‐eight (58) vocally healthy adults were recruited. Ten participants (*n* = 10) could not tolerate scope passage due to narrow nasal anatomy (*n* = 7) or vasovagal response (*n* = 3). Data for an additional 13 participants was incomplete due to either participants requesting early termination (e.g., inability to tolerate the scope, too much gagging reflexes from the laryngoscope, not dependent on laser wattage) (*n* = 12) or recording errors (*n* = 1). The remaining 35 participants (mean age = 27.5 years, SD = 10.3; 29 females, 6 males) with complete data were analyzed. All participants reported no history of voice disorders, laryngeal surgery, or neurological disease, and demonstrated normal voice quality and laryngeal structure on clinical examination. Participant self‐rated voice metrics are presented in Table 1.

**TABLE 1:** Participant self‐rated voice metrics.

Measure (maximum score)	Mean	SD
Vocal effort visual‐analog rating (100)	11.34	16.21
Vocal fatigue visual‐analog rating (100)	8.89	10.58
Vocal tract discomfort visual‐analog rating (100)	4.26	5.41
Vocal tract pain visual‐analog rating (100)	4.43	16.82
VHI‐10 (40)	3.06	3.64
VFI‐part1 (44)	6.83	6.24
VTDS‐total (96)	9.34	8.26

Abbreviations: VFI, voice fatigue index; VHI‐10, voice handicap index 10; VTDS, vocal tract discomfort scale.

### 2.2 Laryngeal Procedures

**FIGURE 1:**
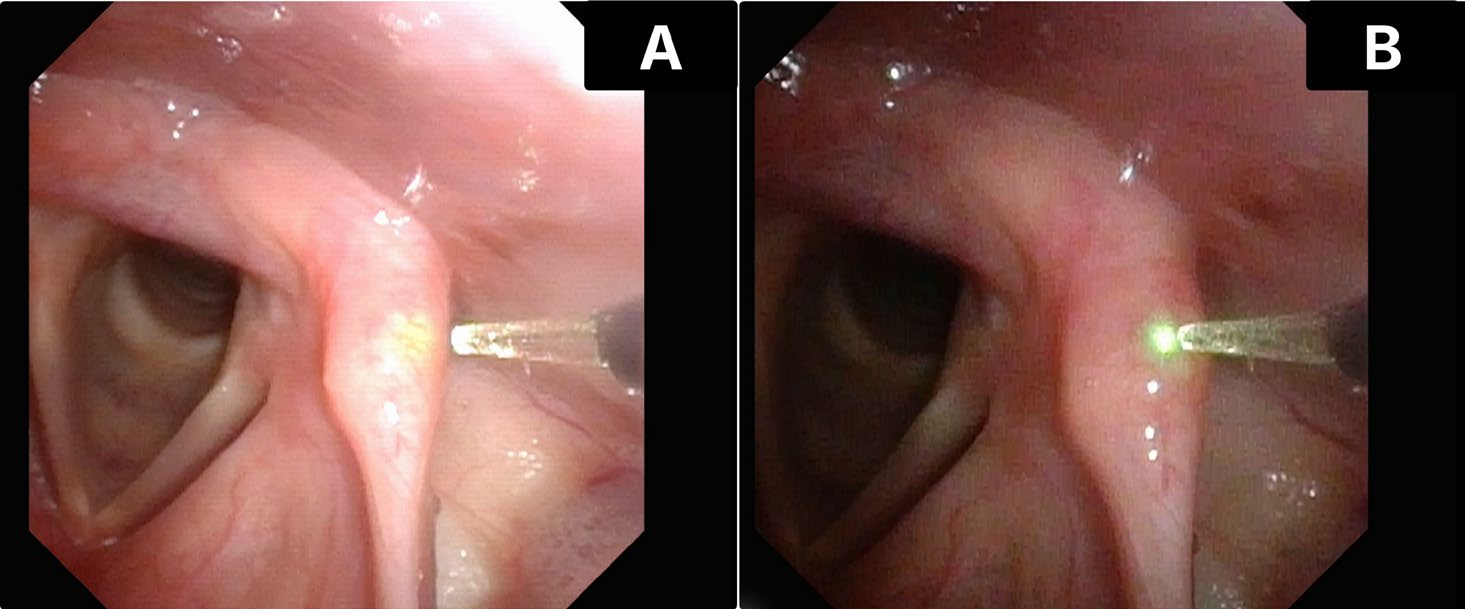
Blue light laser laryngeal sensory testing. (A) Blue light laser with diffuse target; (B) laser with sharp aiming beam in near‐contact mode on the corniculate cartilage/apex of the arytenoid, which is the point at which the laser should be directed and fired.

All three components of laryngeal sensory testing (thresholds, acuity, perturbation) were conducted using a 445‐nm Wolf TruBlue diode laser (Wolf TruBlue, A.R.C. Laser GmbH, Nuremberg, Germany), a 400‐μm fiber, and a channeled laryngoscope (Olympus ENF‐VT2). All laryngoscopy videos were saved for offline analysis (ImageStream Medical nCare v10 at 30 fps, MPEG4 format). A single 30‐ms pulse duration was used for all three procedures based on pilot data demonstrating reliably elicited reflexive laryngeal responses and laryngeal perturbations while remaining below ablative levels. Laser parameters (1–10 W, 30 ms pulse duration) were selected based on pilot testing to remain below ablative thresholds while reliably eliciting sensory and reflexive responses. The side of arytenoid stimulation was randomized for each participant across all three laryngeal sensory testing components. Participants first underwent nasal decongestion and topical anesthesia for 5 min via cottonoid pledgets soaked in oxymetazoline and 4% lidocaine. This allowed anesthesia of the nasal passages without compromising sensory feedback from the larynx. A topical lubricant was placed near the tip of the laryngoscope to maximize participant comfort. The laryngoscope was then passed through the nasal cavity to the pharynx, and the tip of the laser fiber was directed to the corniculate cartilage of the arytenoid until a sharp aiming beam was visualized at 2–4 mm laser‐to‐larynx distance (Figure 1). This achieved near contact with the arytenoid mucosa, with the sharpness of the aiming beam as a gauge. Safety was monitored throughout testing via continuous endoscopic visualization of the stimulation site and participant symptom reporting. The mucosa was inspected following each stimulation for evidence of bleeding, tissue disruption, or other injury, with the intent to stop the protocol, should these presentations occur.

### 2.3 Laryngeal Sensory Threshold Testing

**FIGURE 2:**
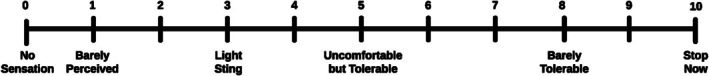
0–10 Likert scale for laryngeal perceptual sensory testing. 0 = no sensation; 10 = maximum sensation.

Laser pulses were always delivered in ascending order from 1 to 10 W (single pulse) to prevent premature induction of laryngeal airway protective reflexes which could heighten sensations and impact subsequent trials [24]. Participants indicated detection after each stimulation by rating perceived intensities on a 0–10 Likert scale (0 = no sensation; 10 = maximum sensation) (Figure 2). Real‐time participant responses were documented by a study team member and checked offline for accuracy using the recorded laryngoscopic videos. Three no‐stimulation “foil” trials (i.e., laser was not fired) were interspersed throughout the 10 trials to identify false positives. Four subjects (~10%) underwent threshold testing twice on two separate days for test–retest reliability (Mean ± SD: 304 ± 275 days). Presence vs. absence of laryngeal airway protection reflexes and other laryngeal guarding behaviors were recorded and analyzed offline.

### 2.4 Laryngeal Discrimination Acuity

Four paired stimulations were randomized from 1–10 W separately for each participant. Each stimulus within a randomized pair was presented sequentially, and participants reported whether the first or second trial felt more intense by raising one or two fingers. The accuracy of participants' ability to detect the more intense stimulation was recorded in real time and checked offline for accuracy.

### 2.5 Laryngeal Perturbations and Acoustic Recordings

Participants sustained the vowel/i/at a comfortable pitch and loudness, each held for 3–5 s, across five trials. During phonation, single 10 W laser pulses (perturbations) were delivered across the trials at random times to elicit transient laryngeal perturbations via airway protection responses (i.e., vocal fold adduction). Nine total perturbations were interspersed across the five trials, some with multiple perturbations within one vocalization trial. The number of perturbations was based on pilot data demonstrating nine trials elicited laryngeal airway protection responses without somatosensory attenuation or saturation, which can occur with multiple repeated stimulations [9], and no tissue ablation beyond mild blanching. No‐stimulations were randomly interspersed throughout the five trials to determine baseline acoustic vocal output measurements. Vocalizations collected during baseline vocal productions and perturbations were recorded in Praat (version 6.4.39) for offline analysis.

### 2.6 Data Processing and Analysis

#### 2.6.1 Laryngeal Adductor Response Scoring

The presence or absence of laryngeal airway protection reflexes were scored by two independent raters using stored laryngoscopy videos. The first rater had 1 year of experience visualizing and analyzing laryngoscopies while the second rater had 5 years of experience. Any disagreements in ratings between the two raters were settled by consensus. Presence of laryngeal airway protection reflexes were determined by brief, ballistic bilateral adduction of the vocal folds with or without presence of ventricular fold activation. The presence or absence of laryngeal guarding behaviors was also noted. Guarding behaviors included vocal folds held in a semi‐adducted state, arrhythmic or ballistic motion of the arytenoids (i.e., laryngeal twitching), and uncontrollable swallowing, gagging, or breath holding (full adduction of the vocal folds for > 0.5 s).

#### 2.6.2 Acoustic Laryngeal Perturbation Analysis

**FIGURE 3:**
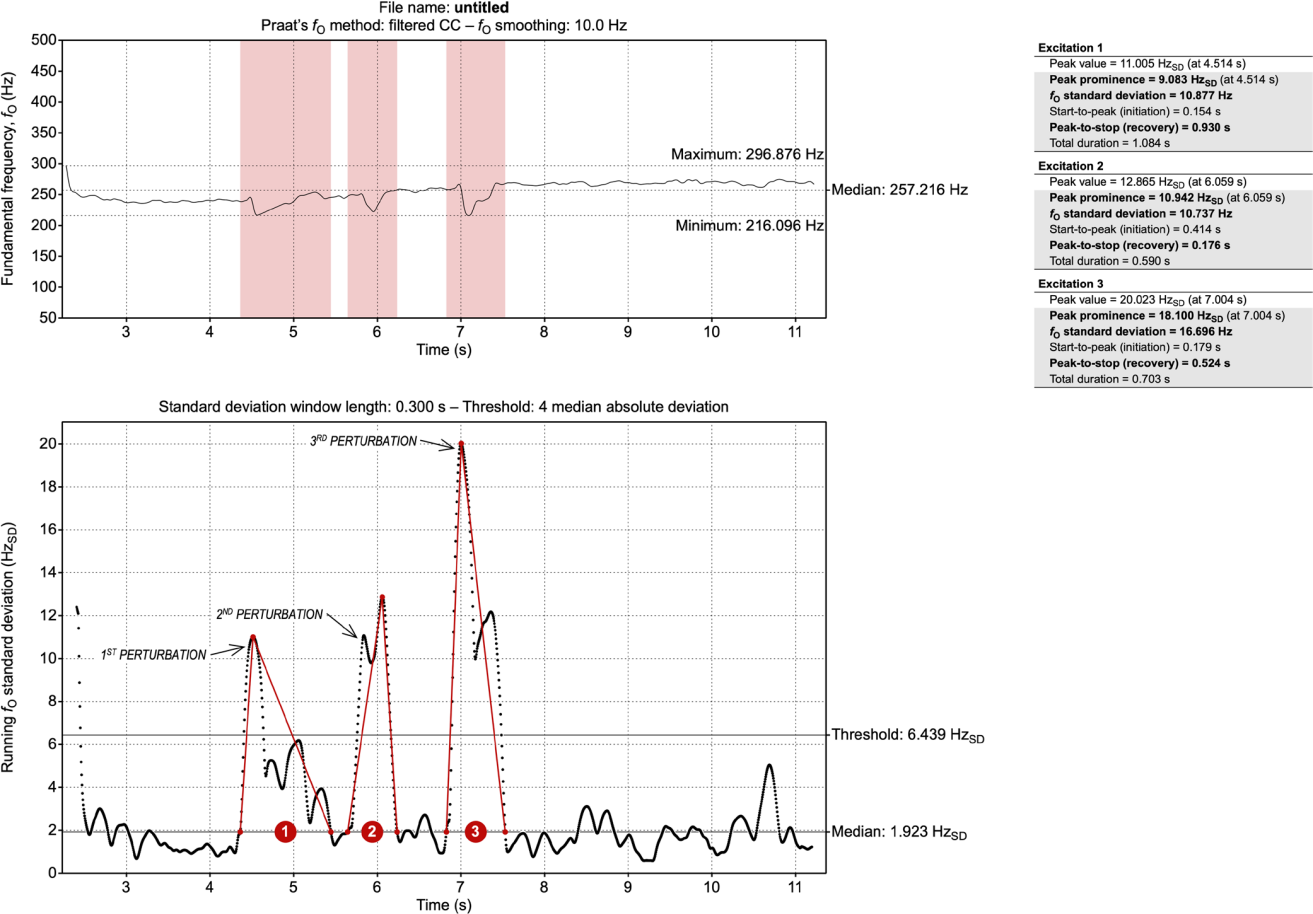
Vocal acoustic output for laryngeal perturbation testing and perturbation testing script output in Praat. Praat script created by Phonanium (https://www.phonanium.com).

Fundamental frequency (f_0_) was extracted in Praat using a filtered cross‐correlation algorithm, interpolated, and smoothed (10 Hz). The script used was customized by Phonanium (https://www.phonanium.com). A 0.3‐s sliding window was used to compute local SD(*f*_0_). Perturbations were automatically identified when SD(*f*_0_) exceeded the median value by ≥ 4 median absolute deviations. Manual adjustments to settings such as the pitch floor, sliding window, and median absolute deviations were completed as necessary to allow the script to accurately capture vocal perturbations. For each perturbation, metrics extracted included: (1) Peak SD(*f*_0_), (2) recovery duration, and (3) local variability during the perturbation window. An example of acoustic vocal output to laryngeal perturbations and analysis output in a representative subject are provided in Figure 3.

#### 2.6.3 Statistical Analysis

Test–retest reliability was assessed using correlations of perceptual scores and repeated measures *t*‐tests of mean laryngeal airway protection thresholds across time points. Mean sensory thresholds during stimulation and foil trials were compared with a repeated measures *t*‐test. Linear mixed‐effects regression (random intercept: participant) examined the relationship between laser wattage and perceived sensory intensity, and logistic regression examined wattage and laryngeal airway reflex presence. Side of stimulation was included as a covariate in both models. Additional logistic regressions tested whether wattage differences predicted discrimination acuity and whether stimulation side affected acuity. Detailed statistical results are provided in the supplement.

## 3 Results

### 3.1 Test–Retest and Rater Reliability of Sensory Testing

The correlation between perceptual ratings across time points was strong and significant (*r* = 0.71, *p* > 0.001), indicating good test–retest reliability. There was also no difference in laryngeal airway reflex thresholds between time points (time point 1 = 4.67, time point 2 = 4.25, *p* = 0.735), indicating reliable elicitation of laryngeal reflexes over time. Reliability between the two independent raters for laryngeal airway reflex scoring was also strong (*κ* = 0.78).

### 3.2 Laryngeal Sensory Thresholds

The mean initial perceptual threshold on the 0–10 Likert scale occurred at 1.46 W (minimum = 1, maximum = 6, SD = 1.17), whereas initial laryngeal airway protection responses first occurred at a mean of 4.62 W (minimum = 1, maximum = 10, SD = 2.04). Across all stimulation levels, participants reported an average laryngeal sensation rating of 4.44 (SD = 2.15), which was significantly higher than sensation ratings during foil trials (*M* = 2.51, SD = 2.13; *p* < 0.001). A significantly greater number of laryngeal airway reflexes occurred above the mean laryngeal response threshold (*n* = 115) than below the mean threshold (*n* = 29; *p* < 0.001, *ω* = 0.60). Sensory perception increased linearly with stimulation intensity, such that each 1‐W increase in wattage was associated with an approximate 0.3‐point increase on the Likert scale (*p* < 0.001), even after controlling for side of stimulation. Additionally, an increase in wattage was associated with a substantially higher likelihood of eliciting a laryngeal airway protection response, with each 1‐W increase corresponding to an approximately 140% increase in odds (*p* < 0.001, OR = 1.38) from baseline. Interestingly, laryngeal airway protection reflexes were observed more frequently with left‐sided stimulation compared to right‐sided stimulation (*p* = 0.026, OR = 0.56). Across the 10 trials, these vocal fold adductory responses occurred in 43% of trials while laryngeal guarding behaviors were present in 55% of trials.

### 3.3 Laryngeal Discriminatory Acuity

The average accuracy of discriminatory acuity across the four pairs of trials was 78.5% (i.e., 3.14/4 pairs). Most of the participants correctly discriminated at least 3 out of 4 trials (46% with 4/4 correct, 26% with 3/4 correct). There were no significant differences in acuity between stimulations delivered to the same arytenoid or different arytenoids (*p* = 0.082) or based on the side of the initial stimulation (*p* = 0.256). Participants were approximately 1.45 times more likely to accurately discriminate between stimulations as the difference in wattage between stimulations increased by 1 W (*p* = 0.002, OR = 1.45).

### 3.4 Laryngeal Perturbations

Peak magnitude of laryngeal perturbations (SD(*f*_0_)) demonstrating the severity of perturbation response across the trials was *M* = 19.35 Hz SD (SD = 18.38, min = 0.95, max = 100). Duration of recovery back to baseline (s) after the laryngeal perturbation was introduced was *M* = 0.48 s (SD = 0.29, min = 0.10, max = 1.30). Variability of the perturbation length within the perturbation window was *M* = 15.43 s (SD = 14.82, min = 0.93, max = 82.28). Sixty‐six percent (65.8%) of total stimulations resulted in auditory or visual perturbations; 79.1% of audible and visual perturbations could be tracked acoustically. Perturbations that could not be tracked were due to laryngeal airway protection reflexes without post‐perturbation vocalizations.

## 4 Discussion

The purpose of this study was to (1) validate a blue‐light laser procedure for assessing laryngeal sensory thresholds, discrimination acuity, and perturbation responses in vocally healthy adults, and (2) develop acoustic methods to quantify these laryngeal perturbations and obtain normative data. This study demonstrates that blue light laser stimulation provides a precise, well‐controlled, and clinically feasible method for laryngeal sensory testing in vocally healthy individuals. The directional stability of the laser's energy allows for reliable stimulation of the arytenoid, a key site of somatosensory reflex initiation. Additionally, SD(*f*_0_)‐based acoustic analysis methods allowed detection and quantification of subtle, transient changes in *f*_0_ stability in response to laryngeal stimulation with the laser.

### 4.1 Feasibility and Reliability of Laryngeal Sensory Testing With the TruBlue Laser

One key benefit of laryngeal sensory testing with the TruBlue laser is that it already exists at many voice centers, making it accessible for laryngeal sensory testing. Laser sensory testing is also well tolerated, as most participants successfully completed testing. Although 16% of participants were unable to tolerate the procedure, this was due to intolerance of the channeled laryngoscope (despite decongestion and lubrication) passing through the nasal cavity because of a small opening in the cartilaginous framework of the nose and not inherent to the laser. While this attrition rate is not negligible, it underscores the inherent challenges and invasive nature of laryngeal assessment, in general. The use of the blue light laser for laryngeal sensory testing is also reliable. Test–retest reliability for threshold testing was statistically significant, and interrater reliability for laryngeal reflex scoring was strong, indicating that the procedure can be performed consistently across individuals and across raters using standardized criteria.

### 4.2 Laryngeal Sensory Perception and Reflexive Laryngeal Airway Protection Responses to Threshold Testing

Participants reported significantly greater perceptual laryngeal sensations during true stimulation trials compared to foil trials, minimizing concern that responses reflected expectancy or generalized discomfort. Participants first reported conscious laryngeal sensation at relatively low stimulation intensities (*M* = 1.46 W), whereas initial laryngeal airway protection reflexes occurred at considerably higher intensities (*M* = 4.62 W). This difference suggests that conscious sensory awareness of laryngeal stimulation precedes reflexive airway‐protective responses, consistent with the notion that perceptual and reflexive pathways, while related, are not functionally equivalent, even in vocally healthy individuals. Sensory perceptual ratings increased linearly with stimulation intensity, and this relationship remained robust even after controlling for stimulation side. In contrast, the likelihood of eliciting a laryngeal reflex response increased nonlinearly, with each 1‐W increase in stimulation associated with a marked increase in odds of reflex activation. The significantly greater number of laryngeal reflex responses occurring above the group mean reflex threshold further supports the construct validity of determining laryngeal thresholds; it also reinforces the role of stimulus intensity as a primary driver of reflexive laryngeal responses.

### 4.3 Laterality Effects in Reflexive Laryngeal Responses to Threshold Testing

The side of stimulation during laryngeal threshold testing significantly influenced laryngeal airway protection reflex occurrence, with left‐sided stimulation eliciting more frequent reflexive responses than right‐sided stimulation. Although the underlying mechanisms for these laterality patterns cannot be elucidated from the present study, a laterality effect could suggest structural differences in the left vs. right recurrent laryngeal nerve [25, 26, 27], asymmetries in vagal innervation [28], or corticobulbar representation [29] of laryngeal function. Interestingly, the linear perceptual–reflexive relationship that was present even after controlling for stimulation side suggests that laterality influences reflexive, but not perceptual sensory processing.

### 4.4 Laryngeal Discrimination Acuity Testing

Participants demonstrated an average discrimination accuracy of nearly 80% across stimulus pairs. Almost half of the participants correctly discriminated all stimulus pairs, indicating that the larynx is capable of fine‐grained sensory discrimination under controlled conditions. Notably, discriminatory accuracy was not affected by whether stimuli were delivered to the same or different arytenoids, nor by the side of stimulation. These findings suggest that discriminatory acuity reflects central sensory processing rather than localized peripheral differences in receptor sensitivity.

### 4.5 Acoustic Consequences of Laryngeal Sensory Perturbations

Continuous measures of local fundamental frequency variability (SD(*f*_0_)) can address gaps in current acoustic analysis by providing high‐resolution and moment‐to‐moment tracking of vocal stability. By using a moving analysis window, SD(*f*_0_) traces can reveal subtle, millisecond‐scale deviations in fundamental frequency that occur in response to the laser pulse. This provides precise quantification of peak magnitude of vocal motor responses to somatosensory laryngeal perturbations to determine severity of laryngeal responses and detailed characterization of initiation and recovery phases within perturbation segments to characterize laryngeal adaptation following perturbations. Stimulation‐induced laryngeal perturbations in this study resulted in reliable acoustic deviations characterized by measurable peak magnitudes (SD(f_0_)) and recovery durations (s). These observed laryngeal responses indicate that laryngeal sensory testing can reliably disrupt ongoing phonation and that these acoustic methods can be used to study laryngeal compensation and adaptation to somatosensory stimuli.

### 4.6 Safety of the Blue Light Laser for Laryngeal Somatosensory Testing

The laser parameters used in this study were selected to remain below tissue‐ablative thresholds. At the highest end of the power range (> 9 W), focal blanching of the mucosa was often noted, but without tissue ablation or bleeding. No participants reported persistent throat pain, dysphonia, airway symptoms, or other delayed complications following the procedure. Importantly, adverse events encountered in the study were related to tolerance of flexible laryngoscopy rather than the laser stimulus itself. These findings suggest that single‐pulse blue light laser stimulation at the parameters used in this protocol represents a low‐risk approach for controlled laryngeal sensory testing in vocally healthy individuals.

### 4.7 Limitations

Several limitations warrant consideration. Although the blue light laser likely produces a localized photothermal stimulus that may activate thermosensitive and nociceptive receptors in the arytenoid mucosa and elicit airway protective reflexes, the specific receptor mechanisms underlying the observed laryngeal responses cannot be determined from this protocol (e.g., thermosensitive, nociceptive, polymodal, and mechanosensitive fibers). Therefore, the present findings should not be interpreted as identifying a specific receptor pathway mediating the elicited laryngeal responses.

Second, while laser testing has more precision over prior sensory testing methods, it still has a finite degree of stimulus delivery uncertainty. Achieving a sharp contour of the aiming beam to get the fiber tip as close as possible to the mucosa consistently will yield the most reliable results. Still, there remains an additional limitation as it relates to the variability of the distance between the laser fiber tip and the mucosal surface. Precise control of fiber‐to‐tissue distance is inherently limited during flexible laryngoscopy due to normal respiratory motion and subtle endoscope movement. As fluence falls with the square of distance, small variations in fiber‐to‐tissue distance alter the fluence to the mucosa. We do not believe this was a major source of error, as the total energy delivered to the target remains the same within the still‐relatively small target area. Nevertheless, we acknowledge that some variability in stimulus intensity likely occurred across trials and participants. While this variability may have contributed to dispersion in sensory and reflex thresholds, the stepwise stimulus paradigm and within‐subject design likely mitigated its impact on overall response patterns. Future studies could improve stimulus precision using fixed‐distance delivery systems or imaging‐based calibration methods to standardize laser‐to‐tissue distance.

Third, although the presence versus absence of laryngeal protective closure reflexes was observed, response latency was not analyzed. Future work involving video capabilities with high temporal resolution like high‐speed laryngoscopy is needed. Future studies that can quantify the magnitude of these protective reflexes (instead of just presence/absence) and then compare reflex response magnitude to magnitude of acoustic measures are also needed to further validate the blue light laser protocol with acoustic vocal output.

## 5 Conclusion

Together, these findings support TruBlue blue light laser stimulation as a low‐risk, well‐tolerated, and clinically accessible method for assessing laryngeal somatosensation and sensorimotor integration. The approach is feasible, reliable, and accurate for measuring perceptual sensitivity, reflexive responses, discrimination acuity, and stimulation‐induced vocal perturbations in vocally healthy individuals. Future research should extend this work to clinical populations with suspected laryngeal sensory or sensorimotor dysfunction (e.g., chronic cough, paradoxical vocal fold motion, laryngeal dystonia, muscle tension dysphonia, and Parkinson's disease) and examine relationships with symptoms such as dysphonia, vocal fatigue, vocal effort, and airway protection. Exploring cognitive and affective influences on laryngeal sensorimotor behavior may also clarify mechanisms of altered integration. Overall, blue light laser stimulation is a promising research tool with potential for future clinical assessment once it has been validated in clinical populations. In the future, this work could lead to a comprehensive tool for clinical assessment and advancing understanding of laryngeal sensorimotor control.

## Funding

This work was supported by NIDCD (R01DC021447) (PI: Adrianna Shembel). The content is solely the responsibility of the authors and does not necessarily represent the official views of the National Institute on Deafness and Other Communication Disorders or the National Institutes of Health.

## Conflicts of Interest

The authors declare no conflicts of interest.

## Supporting information


**Data** **S1:** Shembel et al. Blue Light Laser Testing Supplement: The following statistical terms and abbreviations are used throughout the supplement tables. Test statistics are reported as t(df) for *t*‐tests and regression coefficients, χ^2^(df) for chi‐square tests, and z(df) for logistic regression tests, where df indicates the degrees of freedom associated with the test. *p* values represent the probability of observing the test statistic if the null hypothesis is true. Effect sizes include R, the Pearson correlation coefficient indicating the strength and direction of a linear relationship; d, Cohen's d representing the standardized mean difference between conditions; κ (kappa), Cohen's kappa indicating inter‐rater reliability; and ω, an omega effect size associated with chi‐square tests. Regression models report β (estimate) values representing the change in the outcome associated with a one‐unit increase in the predictor variable, along with odds ratios (OR) in logistic regression, which represent the change in the odds of the outcome for each unit increase in the predictor. Intercept refers to the expected value of the outcome when predictors are at zero or at their reference level. 95% confidence intervals (CI) indicate the range within which the true population parameter is expected to fall with 95% confidence. For mixed‐effects models, percent variance explained represents the proportion of variance accounted for by the predictor relative to an intercept‐only model. Categories shown in parentheses indicate reference categories for categorical predictors.

## Data Availability

The data that support the findings of this study are available from the corresponding author upon reasonable request.
