# Supplementary material for: Validation of the TruBlue Light Laser for Laryngeal Somatosensory and Perturbation Testing
Source: Laryngoscope. 2026 Apr 25;136(9):3841–8. doi: 10.1002/lary.70585 (PMC13460908; doi:10.1002/lary.70585)
Supplement: Supplementary file 1 [file LARY-136-3841-s001.docx]

**Shembel et al: Blue Light Laser Testing**

**Supplement**

Full details for each statistical test are presented in order of in-text appearance

1. Test-retest reliability (Pearson’s correlation)

| Test statistic  (df) | p-value | Effect size | Effect Size 95% confidence interval |
| --- | --- | --- | --- |
| *t*(38) = 6.20 | <.001 | R = 0.71 | 0.51, 0.84 |

2. Reliability of LAR thresholds (repeated measures t-test)

| Test statistic  (df) | *p* value | Effect Size | Effect Size 95% Confidence interval |
| --- | --- | --- | --- |
| *t*(4.403) = 0.36 | 0.736 | d = 0.28 | -1.24, 1.77 |

3. Reliability between raters for LAR scoring (Cohen’s Kappa)

| Result | 95% Confidence interval |
| --- | --- |
| κ = 0.78 | -0.72, 0.84 |

4. Comparing mean laryngeal sensation during stimulation and foil trials (repeated measures t-test)

| Test statistic  (df) | *p* value | Effect Size | Effect Size 95% Confidence interval |
| --- | --- | --- | --- |
| *t*(152.36) = 7.85 | <.0001 | d = 0.90 | 0.67, 1.13 |

5. Comparing number of LARs above and below mean LAR threshold (chi-square goodness of fit)

| Test statistic  (df) | *p* value | Effect Size | Effect Size Confidence interval |
| --- | --- | --- | --- |
| χ² (1) = 51.36**,** | <.001 | **ω** = 0.60 | 0.46, 1.00 |

6. Relationship between sensory perception and stimulation intensity (linear mixed-effects regression)

| Effect | Estimate (β) | Test statistic  (df) | *p* value | % of variance explained over intercept-only model |
| --- | --- | --- | --- | --- |
| Intercept | *2.91* | *t*(52.99) = 9.724 | <.001 | - |
| Watts | 0.29 | *t (313.9) =* 13.08 | < .001 | 34.92 % |
| Side (R) | -0.25 | *t(327.69) = -1.542* | 0.124 | 0.41 % |

Note: categories in brackets represent reference categories

7. Relationship between LAR occurrence and wattage (logistic regression)

| Effect | Estimate | Odds Ratio (OR) | OR 95% confidence interval | Test statistic (df) | *p* value |
| --- | --- | --- | --- | --- | --- |
| Intercept | -1.93 | 0.144 | 0.08, 0.25 | z(336) =  -6.599 | <.001 |
| Watts | 0.32 | 1.38 | 1.27, 1.51 | *z(336)* = 7.05 | < .001 |
| Side (R) | -0.58 | *0.56* | 0.33, 0.93 | *z*(336) = -2.22 | 0.026 |

Note: categories in brackets represent reference categories

8. Relationship between discrimination acuity and side of stimulation (same vs. different side; logistic regression)

| Effect | Estimate | Odds Ratio (OR) | OR 95% confidence interval | Test statistic (df) | P value |
| --- | --- | --- | --- | --- | --- |
| Intercept | 1.04 | 2.83 | 1.78, 4.64 | z(138) = 4.28 | <.001 |
| Side (Same) | 0.82 | *2.27* | 0.94*, 6.14* | z(138) = 1.74 | 0.082 |

Note: categories in brackets represent reference categories

9. Relationship between discrimination acuity and side of stimulation (initial side of stimulation; logistic regression)

| Effect | Estimate | Odds Ratio (OR) | OR 95% confidence interval | Test statistic (df) | *p* value |
| --- | --- | --- | --- | --- | --- |
| Intercept | 1.47 | 4.33 | 2.66, 7.46 | z(138) = 5.61 | <.001 |
| Side (Initial) | -0.49 | 0.62 | 0.27, 1.45 | z(138) = -1.14 | 0.256 |

Note: categories in brackets represent reference categories

10. Relationship between discrimination acuity and difference in wattage between stimulations (logistic regression)

| Effect | Estimate | Odds Ratio (OR) | OR 95% confidence interval | Test statistic (df) | *p* value |
| --- | --- | --- | --- | --- | --- |
| Intercept | -0.34 | 0.71 | 0.28, 1.80 | z(137) = -0.71 | 0.476 |
| Difference in Watts | 0.37 | 1.45 | 1.16, 1.87 | z(137) = 3.09 | 0.002 |
| Side (Same) | 0.79 | 2.20 | 0.88, 6.10 | z(137) = 1.62 | 0.106 |

Note: categories in brackets represent reference categories
